# The Reliability and Validity of Wearable Inertial Sensors Coupled with the Microsoft Kinect to Measure Shoulder Range-of-Motion

**DOI:** 10.3390/s20247238

**Published:** 2020-12-17

**Authors:** Peter Beshara, Judy F. Chen, Andrew C. Read, Pierre Lagadec, Tian Wang, William Robert Walsh

**Affiliations:** 1 Department of Physiotherapy, Prince of Wales Hospital, Sydney, NSW 2031, Australia; judyquick@gmail.com (J.F.C.); mail.andrewread@gmail.com (A.C.R.); 2 Faculty of Medicine, Prince of Wales Clinical School, University of New South Wales, Sydney, NSW 2031, Australia; t.wang@unsw.edu.au (T.W.); w.walsh@unsw.edu.au (W.R.W.); 3 Surgical & Orthopaedic Research Laboratories, Prince of Wales Hospital, Sydney, NSW 2301, Australia; 4 Vald Performance, Brisbane, QLD 4006, Australia; p.lagadec@valdperformance.com

**Keywords:** shoulder, range of motion, sensor, Kinect, goniometry, measurement

## Abstract

Background: Objective assessment of shoulder joint active range of motion (AROM) is critical to monitor patient progress after conservative or surgical intervention. Advancements in miniature devices have led researchers to validate inertial sensors to capture human movement. This study investigated the construct validity as well as intra- and inter-rater reliability of active shoulder mobility measurements using a coupled system of inertial sensors and the Microsoft Kinect (HumanTrak). Methods: 50 healthy participants with no history of shoulder pathology were tested bilaterally for fixed and free ROM: (1) shoulder flexion, and (2) abduction using HumanTrak and goniometry. The repeat testing of the standardised protocol was completed after seven days by two physiotherapists. Results: All HumanTrak shoulder movements demonstrated adequate reliability (intra-class correlation (ICC) ≥ 0.70). HumanTrak demonstrated higher intra-rater reliability (ICCs: 0.93 and 0.85) than goniometry (ICCs: 0.75 and 0.53) for measuring free shoulder flexion and abduction AROM, respectively. Similarly, HumanTrak demonstrated higher intra-rater reliability (ICCs: 0.81 and 0.94) than goniometry (ICCs: 0.70 and 0.93) for fixed flexion and abduction AROM, respectively. Construct validity between HumanTrak and goniometry was adequate except for free abduction. The differences between raters were predominately acceptable and below ±10°. Conclusions: These results indicated that the HumanTrak system is an objective, valid and reliable way to assess and track shoulder ROM.

## 1. Introduction

Measuring the active range of motion (AROM) of the shoulder joint is a fundamental component in any physical examination to diagnose disease, identify functional limitations and monitor patient progress after conservative therapy or surgical intervention [1,2]. Therefore, reproducible measurements of AROM using valid and reliable tools are essential to objectively assess outcomes for individuals with shoulder dysfunction.

Universal goniometry (UG) is the most widely used instrument to measure the range of motion (ROM) of body joints in clinical settings [3,4]. It consists of two transparent arms connected to a circular protractor with scales between 0 and 360 degrees. The centre of the UG is aligned with the centre of the joint, with one arm aligned to the stationary limb and the other arm aligned with the moving limb. The literature recognises UG as a “gold-standard” due to its low cost and portability in clinical settings [5,6,7]. Studies have established modest–excellent intra-rater reliability for UG measurements with intra-class correlations (ICCs) ranging from 0.58 to 0.99 for active abduction [8,9,10,11] and from 0.53 to 0.96 for active shoulder flexion [9,10,11]. However, UG engenders a low inter-rater reliability [1] and requires both hands, thereby reducing the ability to stabilise the joint [12]. Furthermore, UG accuracy is dependent upon clinician skills and experience [13].

The recent development of small, portable, and wearable electromechanical sensors can bridge the gap between the practicality of commonly used clinical tools and more accurate, yet expensive, optical systems. Inertial motion units (IMUs) comprise 3-axis accelerometers, 3-axis magnetometers and 3-axis gyroscopes. IMUs, when paired with data fusion software, can define motion, heading and orientation [14]. The accuracy of wearable IMUs has improved over time with several studies validating its use for joint angle measurement [15,16,17,18,19,20].

Picerno et al. [21] reported excellent intra-rater reliability (ICC = 0.955, 95% confidence interval (CI) = 0.930–0.979) when using a single IMU in 45 healthy subjects and good inter-rater reliability for shoulder flexion (ICC = 0.88) and abduction (ICC = 0.88) in healthy subjects [21,22].

However, one limitation of IMUs is its potential to underestimate large-angle movements and overestimate small-angle movements [16].

The Microsoft Kinect (Microsoft Corp., Redmond, WA, USA) is a low-cost, marker-less system with a depth-sensing camera utilising infrared (IR) laser projection and an IR camera for real-time body tracking in three dimensions (3D). Developers have paired the device with software for the purpose of assessing and measuring human movement. The Kinect has been adapted and evaluated to assess 3-D shoulder kinematics during computer use [23], provide tactile feedback for stroke patients [24] and assess gait in children with cerebral palsy [25]. Several studies have validated the Kinect for the measurement of shoulder joint angles [26,27,28,29,30,31,32].

The Kinect, when compared against the gold-standard Vicon motion capture system for shoulder movements, has demonstrated higher levels of correlation and more acceptable levels of accuracy for forward flexion (Pearson’s correlation (*r)* = 0.993, the limits of agreement (LoA) ±11°, 95% CI: 8.7–12.6°) and abduction (*r* = 0.991, LoA ±11°, 95% CI: 8.7–12.8°) [33]. Additionally, the Kinect has shown good–excellent levels of intra-rater reliability with ICCs between 0.85 and 0.99 for shoulder flexion and ICCs of 0.86–0.98 for abduction [29,30,34,35,36]. Similarly, studies have reported good–excellent levels of inter-rater reliability for Kinect measurements of shoulder flexion (ICCs: 0.89–0.97) and abduction (ICCs: 0.79–0.97) [36,37,38]. The characteristics and reliability values of the studies are summarised in Table 1.

More recently, the integration of IMUs and the Kinect can potentially address the limitations of inertial sensor drift [39], noncalibration [40] and the Kinect’s low sampling frequencies [41]. The sensor fusion provides better visualisation, greater precision and more accurate joint angle measurements [42,43,44].

The HumanTrak system (Vald Performance, Brisbane, Australia) is the coupling of inertial sensors with the updated Microsoft Kinect (v2). Prior studies have validated the Kinect (v2) for the assessment of postural control [41] and for upper limb functional assessment [45]. Mangal et al. [46] compared the Kinect (v2) and goniometry and reported a high correlation for ROM measurements with ICC values ranging between 0.95 and 0.98.

HumanTrak has been validated against the Vicon motion capture system and AMTI force plate system with linear regression results demonstrating excellent correlation for shoulder flexion/extension (average ± standard deviation (SD): 0.907 ± 0.023; minimum: 0.862; maximum: 0.9444) [47].

To our knowledge, no studies have investigated the coupling of inertial sensors with the Kinect to measure shoulder AROM or assess the accuracy of fixed target reaching. Furthermore, previous reliability and validity studies of either of the devices were statistically underpowered due to small sample sizes [27,48,49,50]. Previous systematic reviews have concluded more reliability studies are required to draw stronger conclusions [51,52,53].

Prior to coupled devices being used in clinical practice, they must be assessed for their measurement properties (i.e., reliability and validity). Therefore, this study was designed to determine the intra- and inter-reliability of HumanTrak to measure active free and fixed shoulder motions. To establish construct validity, obtained results were compared with UG, which was chosen for its wide acceptance and applicability in clinical settings [54,55,56,57]. We hypothesised that the coupled system would possess adequate construct validity (ICC: ≥0.70, *r*: >0.75) and reliability (ICC: ≥0.70) for measuring free and fixed shoulder AROMs in healthy individuals.

## 2. Materials and Methods

The aims of the study were threefold: (1) to establish HumanTrak intra- and inter-rater reliability, (2) to determine HumanTrak’s validity with goniometry, and (3) to compare HumanTrak results under two test conditions—free AROM and fixed AROM using consistent targets to reach.

### 2.1. Participants

Fifty asymptomatic adults (27 female and 23 male, mean age: 32.2, mean height: 171.4 cm, left hand dominance: (n = 3)) with no history of shoulder pathology were recruited from a public hospital in Sydney, Australia (Prince of Wales Hospital, Physiotherapy department). Participants from the hospital database were invited to participate via email. All participants gave their written informed consent before participation. The study was approved by the local hospital Ethics Committee (ref no. 17/005) and preregistered in the Australian New Zealand Clinical Registry (ACTRN12617000266369).

The sample size calculation was based on the data from a previous pilot study [58]. The calculation was conducted using the G*Power statistical tool to determine the sample size with 2 raters a significance level of 0.05 and a power of 95%; a sample size of 50 participants would be required.

### 2.2. Raters

Rater A and Rater B were registered Physiotherapists with 10 years and 2 years of clinical experience, respectively. Both raters were responsible for body sensor placement, initiating the protocol instructions and executing the inertial motion system with the Kinect. Rater A was blinded to goniometric measurements between assessments with independent rater-recording results. HumanTrak data were automatically uploaded to a secure cloud-based server, and all ROM values were not visible to raters during or between assessments. The third independent unblinded rater collated and analysed all measurements.

### 2.3. Instruments

#### 2.3.1. HumanTrak

**Figure 1:**
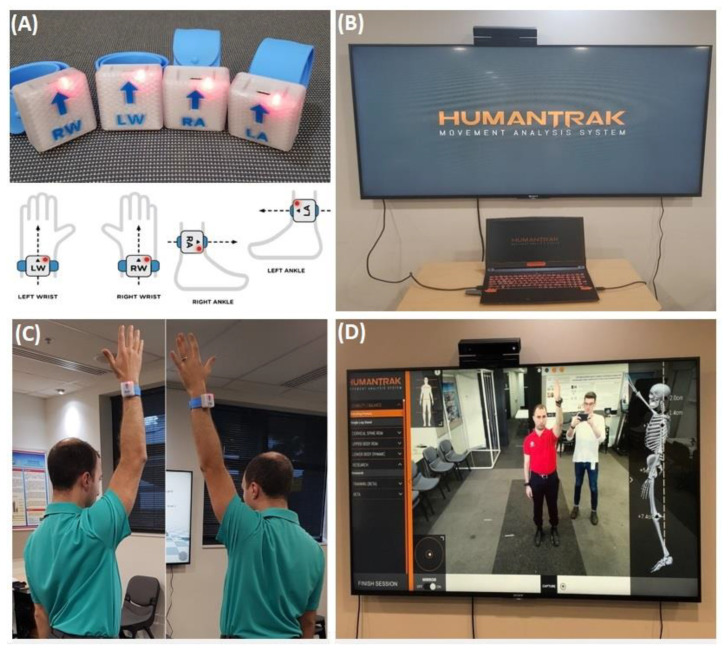
The HumanTrak system. (**A**) Inertial sensors. (**B**) Microsoft Kinect (v2) positioned above a television screen and connected to a laptop. (**C**) Shoulder-free active range of motion (AROM) demonstration. (**D**) HumanTrak interface.

The HumanTrak system integrated four wireless inertial sensors (Figure 1A) worn on the wrists and ankles with an optical sensor (Kinect v2) to estimate human motions (Figure 1B). Optical and inertial data were processed and merged in real time to produce a full-body kinematic model of the subject.

The miniature inertial sensor (dimension: 23 mm × 32.5 mm × 7.6 mm) consisted of a 3-axis gyroscope and a 3-axis accelerometer. Each sensor was applied bilaterally on the dorsal side of the wrist on the ulnar styloid and around the lower leg above the lateral malleolus. The sensor was securely attached to a plastic-covered slap bracelet with an interior metal coil spring.

A high-speed radio frequency (RF) protocol transferred the inertial data to a receiving computer. The automatic calibration of the system was established, and the Kinect was positioned at a height of 1.4 m with a tilt of −2.0°. Feet markers were placed 1.5 m away from the Kinect to ensure consistency with the participant placement.

#### 2.3.2. Goniometer

A single 12 inch plastic goniometer, model 12-1000 (BASELINE^®^, Boise, ID, USA), was used by Rater A for all shoulder measurements.

### 2.4. Procedures

**Figure 2:**
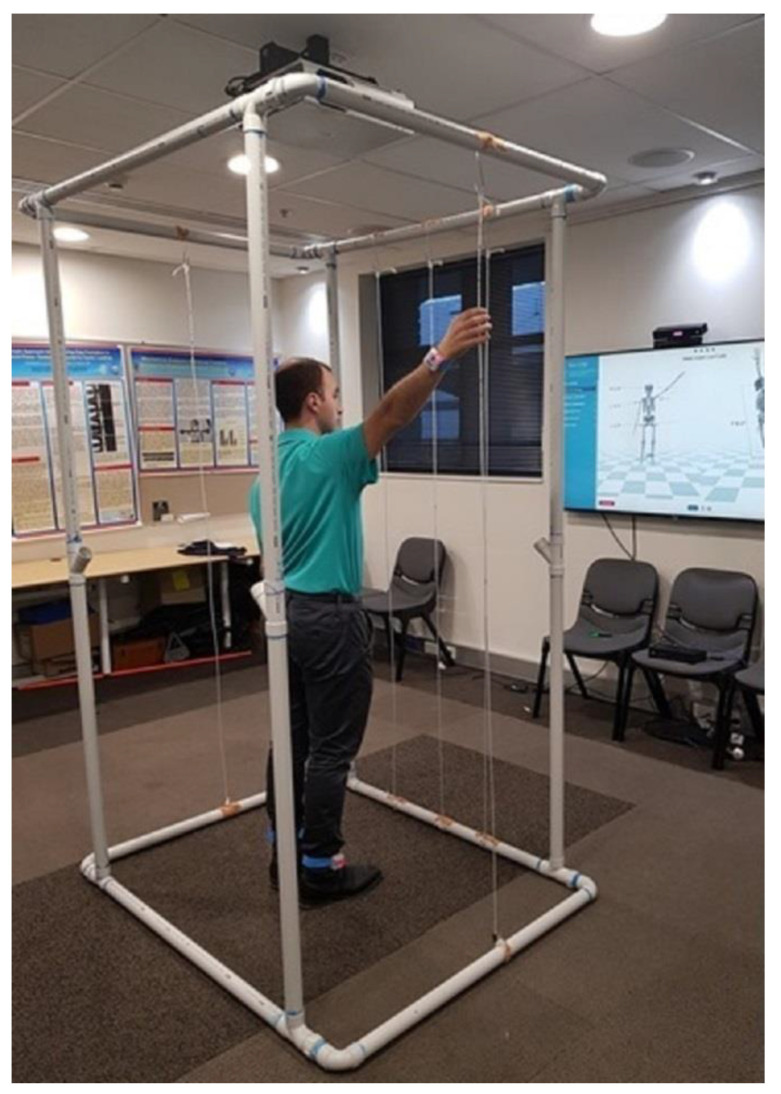
Fixed AROM with a freestanding cage.

Bilateral shoulder measurements of forward flexion and abduction were performed in the standing position (Figure 1C). Instructions were verbally standardised, and participants were asked to move their arms “as far as possible” for free peak AROM. To assess fixed AROM, participants stood inside a PVC-constructed freestanding cage with coloured felt-tip-marked suspended ropes denoting the standardised position to reach in flexion and abduction (Figure 2). Targets were determined by the participant’s height and comfort level. Identical targets were recorded and used by both raters on the second assessment. All shoulder movements were performed for three repetitions with a 3 s hold.

**Figure 3:**
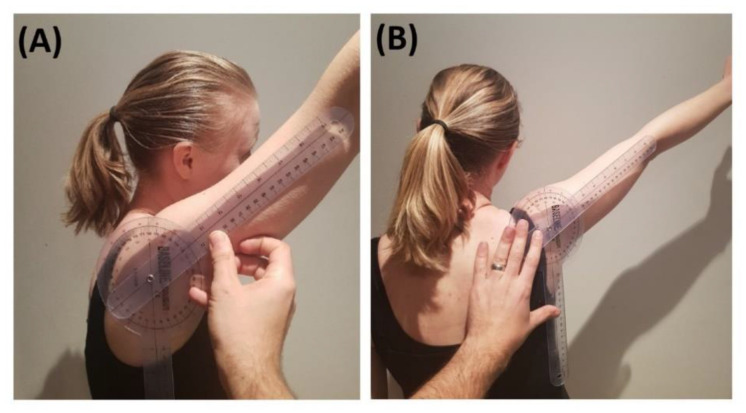
Goniometry measurement. (**A**) Forward flexion. (**B**) Abduction.

Rater A assessed free peak AROM with a universal goniometer using the same protocol as that by Jain et al. [59]. The forward flexion angle was measured by placing the fulcrum of the goniometer on the middle of the glenoid fossa, one arm of the goniometer being aligned with the lateral epicondyle of the humerus and the other arm on a vertical line in the coronal plane (Figure 3A). The abduction angle was defined by placing the fulcrum of the goniometer in the middle of the posterior glenohumeral joint line, one arm of the goniometer being aligned with the lateral epicondyle of the humerus and the other arm on a vertical line in the sagittal plane (Figure 3B).

### 2.5. Data Capture and Processing

**Figure 4:**
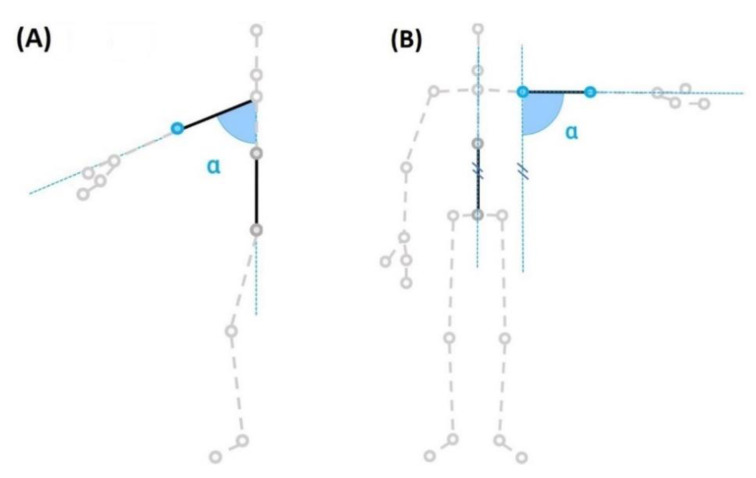
Shoulder joint range-of-motion (ROM) calculation with HumanTrak. (**A**) Shoulder forward flexion: angle (α) between the trunk (mid spine-spine base) and the upper limb (shoulder-elbow) in the sagittal plane. (**B**) Abduction: angle (α) between the trunk (mid spine-spine base) and the upper limb (shoulder-elbow) in the frontal plane.

The HumanTrak software captured data in real time at 100 Hz. The positional 3-D data of the kinematic model was postprocessed using a two-way Butterworth filter of order four to remove any residual noise, prior to calculating AROM values. Shoulder flexion/extension was calculated in the sagittal plane as the angle between the shoulder-elbow axis and the spine vertical axis (Figure 4A). Shoulder abduction/adduction was calculated in the coronal plane as the angle between the shoulder-elbow axis and the spine vertical axis (Figure 4B).

### 2.6. Statistical Analysis

Data analysis was performed with SPSS version 24 for Windows. Descriptive data including mean measurement angles with SDs were calculated for Rater A’s intra-rater analysis.

The reliability of all measurements was determined by ICC Model 3, 1 for the intra-rater component of analysis. This model was used, as the single rater was the only rater of interest. An (ICC) Model 2, k was used for the inter-rater analysis to determine if HumanTrak can be used with confidence and reliability among equally trained clinicians [60,61].

ICCs for absolute agreement were reported, and the mean value from each testing session was used for the analysis.

Given that ICCs may be affected by a limited range of data or intersubject variability, absolute measures of reliability were determined [62]. The standard error of measurement (SEM) was calculated using the equation: SEM = SD × $\sqrt{1-\mathrm{ICC}}$. From the SEM, the minimal detectable change (MDC) was calculated using the formula MDC = 1.65 × SEM × $\sqrt{2}$ for test-retest data. This clinically meaningful degree of difference determines the magnitude of change that would exceed the threshold of measurement error [63]. Inter-rater reliability was visualised using Bland–Altman plots [64] with 95% limits of agreement (LoA) calculated using the formula: 95% LoA = mean difference ± 2SD. There is currently no agreed criterion in the literature for the acceptable levels of inter-observer agreement. Based on prior studies and clinical experience, any differences between raters greater than ±5° were considered clinically significant, and exceeding ±10° were considered unacceptable [65]. Construct validity was determined with an (ICC) Model 3, k, Bland–Altman plots, mean differences, 95% LoA and Pearson’s correlation to determine if both methods of measurement analysis produced comparable results. If the 95% LoA were greater than ±5°, then the discrepancies between measurement systems were considered clinically significant [27,64].

### 2.7. Quality Criteria

Reliability and construct validity were assessed using the criteria by Terwee et al. [66]. Reliability was considered adequate, if the tests ICC was ≥0.7, with an ICC of <0.7 considered inadequate. For construct validity, a correlation of ≥0.75 was considered adequate, and that of <0.75 was considered inadequate. These criteria have been validated in previous studies of measurement properties [67].

## 3. Results

### 3.1. Intra-Rater Reliability

The results of intra-rater reliability with ICC, SEM and MDC values are presented in Table 2. All free and fixed AROM HumanTrak shoulder movements demonstrated adequate reliability (ICC ≥ 0.7).

For free AROM, the highest intra-rater reliability was observed for forward flexion. For fixed AROM in the cage, the highest intra-rater reliability was observed for abduction. All SEM and MDC values were relatively low, indicating good absolute reliability.

Adequate correlations (*r* ≥ 0.75) between week 1 and week 2 for HumanTrak were demonstrated for free forward flexion and fixed abduction (Table 2).

Goniometric intra-rater reliability measurements are presented in Table 3. All fixed and free AROM movements demonstrated adequate intra-rater reliability with the exception of free abduction.

### 3.2. Inter-Rater Reliability

The results of inter-rater reliability with ICC, SEM and MDC values are presented in Table 4. For free AROM, all movements demonstrated adequate inter-rater reliability (ICC > 0.70). For fixed AROM in the cage, adequate inter-rater reliability was only observed for abduction.

**Figure 5:**
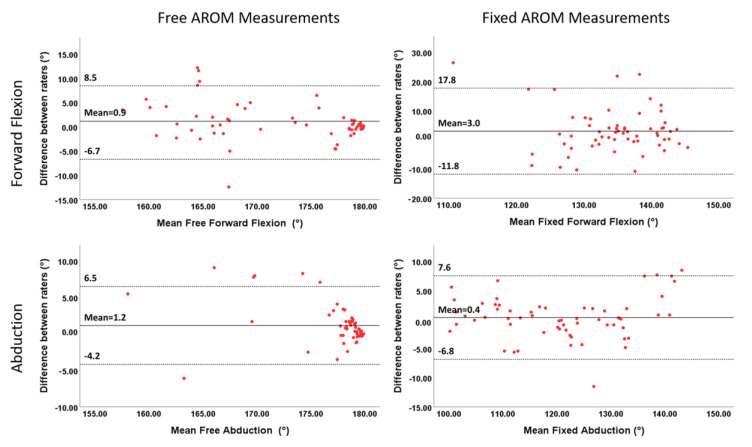
HumanTrak differences between raters for free and fixed forward flexion and abduction AROM. The solid black line is the mean difference between raters, and the segmented black lines show the 95% limits of agreements.

To analyse differences further, agreement was assessed using Bland–Altman plots (Figure 5). The mean differences between raters were 0.9° (free forward flexion), 1.2° (free abduction), 3.0° (fixed forward flexion) and 0.4° (fixed abduction).

The limits of agreement determined by the 95% CI were from –6.7° to 8.5° for free forward flexion and from –4.2° to 6.5° for free abduction. Fixed AROM differences varied between –11.8° and 17.8° for forward flexion and between –6.8° and 7.6° for abduction. For all movements, differences were predominately below ±10°, and the majority of values did not exceed the clinically significant difference ±5°.

### 3.3. Construct Validity

Table 5 shows the correlation between HumanTrak and UG. HumanTrak demonstrated adequate agreement only for free forward flexion. In contrast, the fixed AROM measurements demonstrated adequate correlations for both shoulder movements.

The 95% limits of agreement ranged between −11.3° and 15.4° for free forward flexion, between −15.6 and 21.7° for free abduction, between −4.8 and 4.8° for fixed forward flexion and between −7.8 and 12.1° for fixed abduction.

**Figure 6:**
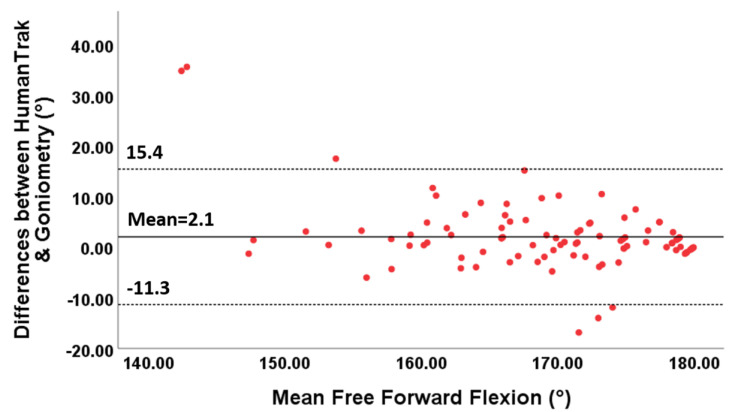
Bland–Altman plots for free forward flexion with limits of agreement. The differences between the two devices are plotted on the Y-axis, and the mean scores are plotted on the X-axis.

**Figure 7:**
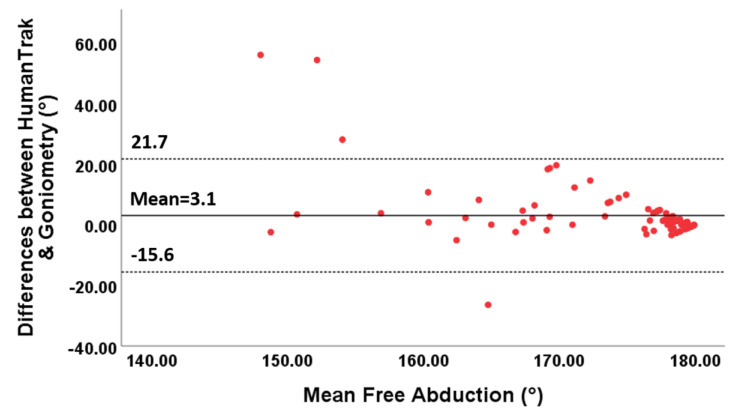
Bland–Altman plots for free abduction with limits of agreement. The differences between the two devices are plotted on the Y-axis, and the mean scores are plotted on the X-axis.

Furthermore, the points in the Bland–Altman plots for free forward flexion (Figure 6) and abduction (Figure 7) were uniformly and tightly scattered around the horizontal axis.

**Figure 8:**
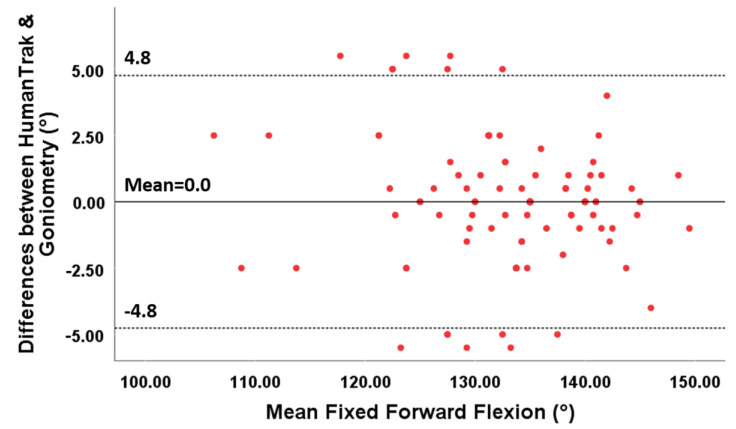
Bland–Altman plots for fixed forward flexion with limits of agreement. The differences between the two devices are plotted on the Y-axis, and the mean scores are plotted on the X-axis.

**Figure 9:**
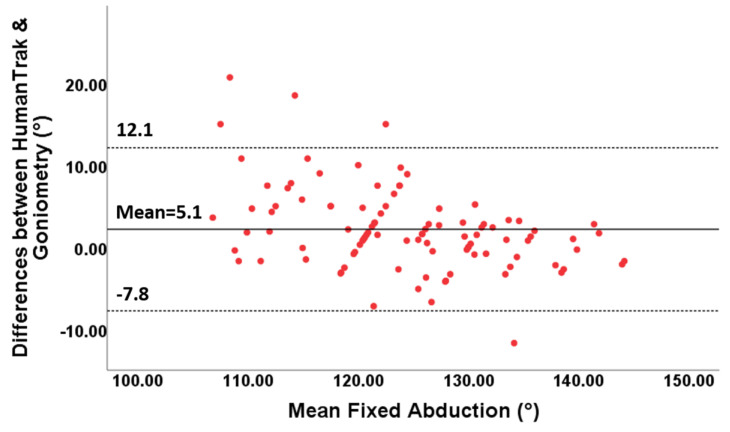
Bland–Altman plots for fixed abduction with limits of agreement. The differences between the two devices are plotted on the Y-axis, and the mean scores are plotted on the X-axis.

For fixed AROM, the points on the Bland–Altman plots demonstrated variability around the mean constant (Figure 8 and Figure 9). Small mean differences between the two methods for forward flexion and abduction were demonstrated (Table 5).

## 4. Discussion

Accurate and reliable measurements of shoulder mobility are important to diagnose pathological conditions, evaluate treatment effectiveness and monitor progress after surgery. This study demonstrates that a system using wearable inertial sensors coupled with the Microsoft Kinect is a reliable and valid way to measure active shoulder forward flexion and abduction.

The results confirmed the hypothesis that, under both test conditions, HumanTrak had higher intra-rater reliability for shoulder forward flexion (ICC = 0.93 and 0.81) and abduction (ICC = 0.85 and 0.94) than UG. HumanTrak demonstrated lower SEM and MDC values than goniometry with small measurement errors (2–4°), indicating good absolute reliability. Similar to our results, an initial study utilising the BioCap system (the beta version of HumanTrak) reported adequate intra-rater reliability for free shoulder forward flexion (ICC = 0.93 and 0.83) and abduction (ICC = 0.82) [58]. Previous studies have only investigated inertial sensors or the Kinect separately to measure shoulder joint angles. Using an electromagnetic tracking system (FASTRAK), Jordan et al. [68] achieved adequate intra-rater reliability for measuring active shoulder flexion (ICC = 0.74). Huber et al. [27] reported excellent intra-rater reliability (ICCs: 0.76–0.98) with the Kinect in a small sample size of 10 healthy adults.

This study examined the inter-rater reliability of inertial sensors coupled with the Kinect. All free and fixed AROM movements demonstrated adequate inter-rater reliability (ICCs = 0.88–0.98) with the exception of fixed forward flexion (ICC = 0.65). The Bland–Altman plots demonstrated acceptable levels of agreement between raters, and the 95% LoA were below ±10° for all movements, except for fixed forward flexion. By constructing a freestanding cage with suspended ropes, we used a novel approach to assess HumanTrak’s accuracy in detecting the same fixed point. Despite the best efforts to ensure the same targets were reached within and between trial sessions, human error from the participant cannot be discounted. Therefore, the higher SEM and MDC values for fixed forward flexion were likely due to experimental errors from participants not reaching the same target between raters.

This study established adequate reliability and construct validity between HumanTrak and goniometry only for free forward flexion (ICC = 0.84, *r* = 0.77). Similarly, Lee et al. [49] reported excellent agreement with the Kinect and goniometry for active shoulder flexion (ICC = 0.86) and abduction (ICC = 0.93). Although authors reported higher ICC values, the study had a significantly smaller sample size of 15 healthy volunteers.

The Bland–Altman plots demonstrated low mean differences and wide 95% LoA above the clinically significant threshold of ±5°. For agreement, clinicians should recognise differences between HumanTrak and goniometry can be expected to vary by up to ±15°. Compared to our results, Huber et al. [27] reported wider limits of agreement (95% LoA exceeding ±30°) and significant levels of bias between the Kinect and goniometry for measuring active shoulder ROM. In contrast, Yoon et al. [48] reported acceptable validity between IMU and goniometry for shoulder angle measurements with the 95% LoA discrepancies between the measurements ranging within ±5°. However, the sample size was small, participants were predominately women, and all shoulder movements were passive.

Experimental limitations may explain why the highest intra- and inter-rater reliability values were achieved for free forward flexion and fixed abduction. Participants performing free forward flexion without a target were more likely to consistently move their arm in the single sagittal plane. In contrast, it was observed that reaching forward for a target in the cage elicited substitute movements involving excessive shoulder abduction. It was observed that fixed abduction demonstrated higher reliability than free abduction. An explanation for this result is that unrestricted abduction can be performed in the scapular or coronal plane, resulting in a less consistent measurement. These findings highlight that regardless of HumanTrak’s accuracy and reliability, careful positioning and rater observation are essential to avoid compensatory or unwanted movements.

We acknowledge this study had several limitations. First, we used goniometry as a “gold standard” rather than a motion capture system. Authors chose to not replicate findings that have previously validated HumanTrak against the Vicon system [47]. While the Vicon system is widely accepted as the gold standard for human motion analysis [69] and has demonstrated acceptable levels of accuracy when compared with the Kinect sensor for measuring shoulder ROM in healthy volunteers [18], it is an onerous and expensive system to implement in clinical settings.

Participants in this study were healthy, predominately young, and not stratified for age. However, this population was appropriate, since we eliminated functional deficits and pain as potential confounding variables to establishing reliability. For practical reasons, no attempts were made to measure participants at the same time of day on each occasion; hence, we did not control for any potential variations in AROM at different times of the day. Finally, caution should be used in interpreting the HumanTrak inter-rater reliability results due to the smaller sample size of 15 participants.

Current system limitations reside in the HumanTrak system calibration process where each subject is required to stand straight to allow adjustments for any tilt in camera orientation. Slight errors in tilt calibration can occur in cases where the participant is unable to completely stand straight. However, this can be avoided if a healthy participant is initially used for calibration prior to tracking the rest of the participants, since the tilt calibration is only camera-dependent and will not change between participants.

Shoulder AROM was calculated into specific planes: sagittal plane for flexion/extension and coronal plane for adduction/abduction. These planes are defined from the camera sensor’s reference. As such, a current limitation is that the subject is required to perfectly face the camera to maximise the accuracy of the ROM measurements. Errors in measurement might occur, if the subject is facing the camera at an angle, with the amount of errors increasing as the angle increases.

## 5. Conclusions

HumanTrak is a reliable and valid tool for surgeons and physiotherapists to deliver a quick and functional evaluation of the shoulder. As a marker-less, nonintrusive system for ROM evaluation, HumanTrak demonstrates potential as feasible adjunct to clinical observation in rehabilitative and outpatient clinical settings. Inertial sensor technology coupled with optical-based systems can provide accurate and objective data to support clinical decision-making and facilitate better clinical outcomes.

## Figures and Tables

**Table 1:** Summary of Kinect reliability studies.

Study	Target Population (*n*)	Intra-Rater Reliability ICC		Inter-Rater Reliability ICC	
		Shoulder Flexion	Shoulder Abduction	Shoulder Flexion	Shoulder Abduction
Hawi et al.	Healthy, free ROM without deficits (*n* = 7)	0.99	0.96	-	-
Bonnechère et al.	Healthy (*n* = 48)	-	0.73	-	-
Çubukçu et al.	Healthy (*n* = 40)	0.851	0.861	-	-
Hwang et al.	Wheelchair usage for 1 year, able to sit upright for at least 4 h a day, use a wheelchair for >40 h/week (*n* = 8)	L = 0.96 R = 0.92	L = 0.92 R = 0.96	-	-
Da Cunha Neto et al.	Healthy (*n* = 10)	0.97	0.98	0.91	0.97
Guneysu et al.	Healthy, children aged 3–11 (*n* = 8)	-	-	0.8961	0.7935
Milgrom et al.	Spinal cord injury, ability to self-propel a manual wheelchair, wheelchair usage for at least 75% of daily activities (*n* = 5)	-	-	0.97	0.94

ICC = intra-class coefficient, ROM = range of motion; *n* = number.

**Table 2:** Intra-rater reliability and Pearson’s *r* (HumanTrak) of rater A (*n* = 50).

Free AROM	Mean ± SD (°)	ICC_3,1_	95% CI	SEM (°)	MDC (°)	*r*
Forward flexion	169.7 ± 8.4	0.93	0.89–0.96	2.2	6.1	0.89
Abduction	175.8 ± 6.8	0.85	0.77–0.90	2.7	7.5	0.73
**Fixed AROM**						
Forward flexion	134.8 ± 8.2	0.81	0.72–0.87	3.6	10.0	0.69
Abduction	124.5 ± 10.8	0.94	0.91–0.96	2.7	7.5	0.91

SD = standard deviation; ICC = intra-class correlation coefficient; CI = confidence interval; SEM = standard error of measurement; MDC = minimal detectable change; r = Pearson’s correlation.

**Table 3:** Intra-reliability and Pearson’s *r* (Goniometry) of rater A (*n* = 50).

Free AROM	Mean ± SD (°)	ICC_3,1_	95% CI	SEM (°)	MDC (°)	*r*
Forward flexion	168.5 ± 10.3	0.75	0.64–0.82	5.2	14.4	0.75
Abduction	172.5 ± 10.8	0.53	0.38–0.66	7.4	20.5	0.53
**Fixed AROM**						
Forward flexion	132.5 ± 9.2	0.70	0.50–0.82	5.0	13.9	0.58
Abduction	124.4 ± 8.2	0.93	0.88–0.96	2.7	7.4	0.87

SD = standard deviation; ICC = intra-class correlation coefficient; CI = confidence interval; SEM = standard error of measurement; MDC = minimal detectable change; r = Pearson’s correlation.

**Table 4:** HumanTrak Inter-rater reliability (*n* = 15).

**Free AROM**	**ICC_2,k_**	**95% CI**	**SEM (°)**	**MDC (°)**
Forward flexion	0.92	0.87–0.96	2.0	5.6
Abduction	0.88	0.77–0.93	1.5	4.3
**Fixed AROM**	**ICC_2,k_**	**95% CI**	**SEM (°)**	**MDC (°)**
Forward flexion	0.65	0.41–0.80	4.6	12.7
Abduction	0.98	0.96–0.99	1.9	5.1

ICC = intra-class correlation coefficient; CI = confidence interval; SEM = standard error of measurement; MDC = minimal detectable change.

**Table 5:** Correlation between HumanTrak and goniometry (*n* = 50).

**Free AROM**	**Mean ± SD (°)**	**ICC**	**95% CI**	**Mean diff (°)**	***r***
Forward flexion	169.4 ± 9.5	0.84	0.72–0.87	2.05	0.77
Abduction	174.5 ± 9.1	0.59	0.60–0.82	3.05	0.50
**Fixed AROM**	**Mean ± SD (°)**	**ICC**	**95% CI**	**Mean diff (°)**	***r***
Forward flexion	133.0 ± 8.2	0.98	0.97–0.99	0.00	0.96
Abduction	124.3 ± 9.4	0.91	0.84–0.94	2.18	0.87

ICC = intra-class correlation coefficient; CI = confidence interval; diff = difference; *r* = Pearson’s correlation.
